# Hantavirus surveillance and genetic diversity targeting small mammals at Camp Humphreys, a US military installation and new expansion site, Republic of Korea

**DOI:** 10.1371/journal.pone.0176514

**Published:** 2017-04-27

**Authors:** Heung-Chul Kim, Won-Keun Kim, Terry A. Klein, Sung-Tae Chong, Peter V. Nunn, Jeong-Ah Kim, Seung-Ho Lee, Jin Sun No, Jin-Won Song

**Affiliations:** 1 5th Medical Detachment, 168th Multifunctional Medical Battalion, 65th Medical Brigade, United States of America; 2 Department of Microbiology, College of Medicine, Korea University, Seoul, Republic of Korea; 3 MEDDAC-Korea, 65th Medical Brigade, United States of America; Virginia Commonwealth University, UNITED STATES

## Abstract

Small mammal surveillance was conducted (2008–2010, 2012) at Camp (Cp) Humphreys, a US Army installation and new expansion site, Republic of Korea (ROK), to identify hemorrhagic fever with renal syndrome health threats to US military/civilian populations during its ongoing expansion phase. Small mammals were collected using Sherman live capture traps and transported to Korea University where they were euthanized, tissues removed, and assayed to determine hantavirus IgG antibody-positive and hantavirus-positive rates by RT-PCR. A total of 2,364 small mammals were captured over 11,300 trap nights (capture rate = 20.92%). *Apodemus agrarius* was the most commonly collected (76.65%), with capture rates of 9.62% and 21.70% for Cp Humphreys and the expansion site, respectively. Overall, Hantaan virus (HTNV) IgG antibody-positive (Ab+) rate for *A*. *agrarius* was 2.15% (39/1,812). A total of 5.43% (10/184) *Crocidura lasiura*, 0.79% (2/254) *Microtus fortis* and 2.44% (1/41) *Micromys minutus* were serologically IgG Ab+ for hantaviruses. HTNV-specific RT-PCR demonstrated that 28.2% (11/39) HTNV Ab+ *A*. *agrarius* harbored the 328-nt sequence of the G_C_ glycoprotein-encoding M segment of HTNV. Among them, the whole genome sequences of 3 HTNV strains were obtained by conventional RT-PCR and Rapid Amplification cDNA Ends PCR. Phylogenetic analyses of the HTNV strains from Cp Humphreys and the expansion site, Pyeongtaek, show a greater diversity of rodent-borne hantaviruses compared to HTNV previously identified in Gyeonggi province of the ROK. Thus, this study provides significant insights for raising HFRS threat awareness, analysis, and risk reduction strategies in southern Gyeonggi province.

## Introduction

Hantaan (HTNV) and Seoul viruses (SEOV), etiologic agents of hemorrhagic fever with renal syndrome (HFRS) in the Old World, pose serious health threats to military and civilian personnel residing, working, or conducting routine military operations in rodent-infested environments. HTNV, transmitted through the inhalation of dusts containing HTNV-infected *Apodemus agrarius* excreta, poses the greatest threat due to its extended incubation period (up to 50 days), mean duration of illness from onset of symptoms to complete recovery, and overall morbidity and mortality rate of 9.46% reported for US military personnel in the presence of quality medical management [1–4]. The morbidity of SEOV ranges from mild to moderate and mortality rate <1%, is similarly transmitted by inhalation of dusts containing SEOV-infected *Rattus norvegicus* excreta, and while much less severe than HTNV infections, poses a threat for personnel occupying or conducting operations in rat infested urban environments [5].

In 2000, the 18^th^ Medical Command launched a comprehensive rodent-borne disease surveillance aimed primarily to assess HFRS risks based on activities of military personnel, habitat characteristics, relative rodent population densities, and HTNV IgG antibody-positive (Ab+) at United States (US) and Republic of Korean (ROK) operated military training sites [4,6–10]. Rodent-borne disease surveillance at training sites near the demilitarized zone (DMZ) showed that *A*. *agrarius* populations, based on trap rates, remained relatively stable and that high gravid rates were observed in August-September, which correlated with increased numbers of HFRS cases from late September-December [4,7,9,10]. The proposed consolidation of US military forces to Camp (Cp) Humphreys resulted in the purchase of adjoining farmlands (rice paddies and associated irrigation and drainage systems). By 2008, the area immediately adjacent to the preexisting Cp Humphreys boundary was under development (land graded and paddies filled) to make way for the construction of roads, drainage systems, housing, and other structures that support military operations and other related activities. From 1986–2016, when detailed epidemiological data were available, there were no reported HFRS cases attributed to exposure at Cp Humphreys and nearby local training areas. However, due to landscape modifications and construction activities that may alter HFRS risks, a comprehensive rodent-borne disease surveillance program was conducted at the Cp Humphreys and the new expansion site. HFRS risk factors were estimated based on associated human activities that considered environmental, biological, and hantavirus prevalence associated with rodents and soricomorphs for both the established cantonment area of Cp Humphreys and the fallow farmlands that were being graded/filled that may alter HFRS risks. This report focuses on epidemiological data that identifies environmental factors, seasonal rodent population densities, biological factors, and HTNV serological and molecular prevalence related to HTNV transmission risks for Cp Humphreys and the expansion site, Pyeongtaek. The whole genomic sequences of HTNV newly obtained for Cp Humphreys and the expansion site, Pyeongtaek, were phylogenetically characterized demonstrating a greater diversity of rodent-borne hantaviruses.

These data are useful for developing local HFRS disease threat awareness, analysis, and risk reduction strategies for civilians and military personnel in southern Gyeonggi province, ROK.

## Materials and methods

### Ethics statement

All trapping of small mammals was approved by US Forces Korea (USFK) in accordance with USFK Regulation 40–1 “Prevention, Surveillance, and Treatment of Hemorrhagic Fever with Renal Syndrome” at US Military Installations and US and ROK Operated Military Training Sites [11]. Standard procedures were followed for the collection and transportation of specimens to minimize hazards from potentially infected animals as previously described and all personnel processing animals at the Korea University laboratory were vaccinated using a Korean-approved Hantavirus vaccine (Hantavax^®^) [12, 13]. Small mammals were euthanized by cardiac puncture under isoflurane anesthesia in strict accordance with the Korea University Institutional Animal Care and Use Committee (KUIACUC, #2010–212) protocol approved for this study. All efforts were made to minimize suffering.

### Site description

**Fig 1:**
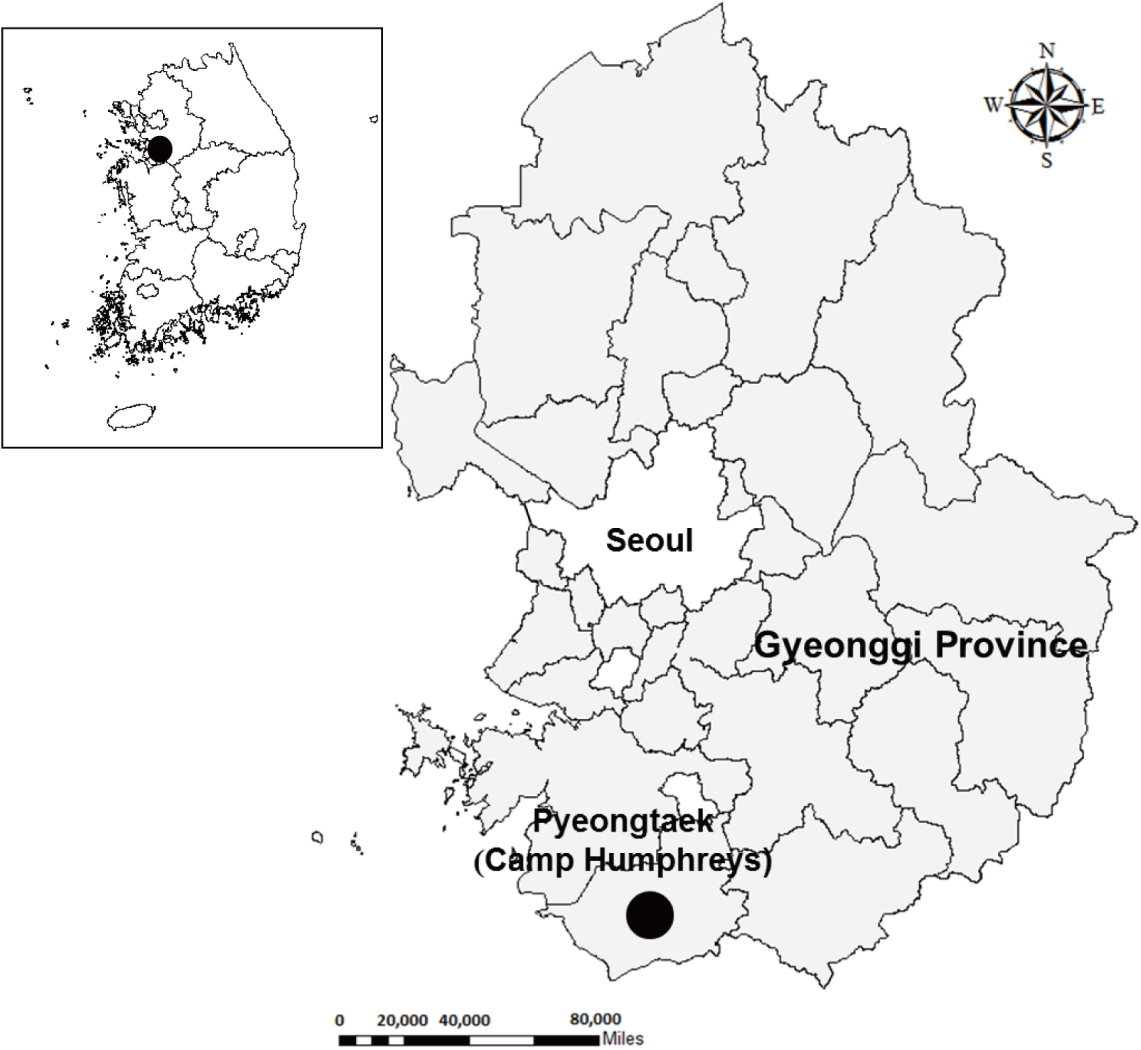
Location of Camp Humphreys, Gyeonggi province, Republic of Korea where rodent-borne disease surveillance was conducted from 2008–2010 and 2012.

Cp Humphreys, located near Pyeongtaek in southern Gyeonggi Province, was designated as a US military hub incorporating US military installations near the DMZ and elsewhere **(Fig 1)**. The areas surveyed included: (1) Cp Humphreys (hereafter referred to as “Cp Humphreys”) and (2) the new expansion site (referred to hereafter as “expansion site”) for the construction of structures for military operations and housing and development of roads, drainage systems, and outdoor recreational areas.

Cp Humphreys is bounded by a small town (Anjeong-ri, Paengseong-eup), farmland, and the expansion site. It consists of an airstrip, asphalt roads, and structures for military operations and housing, streams, man-made water impoundments, and drainage ditches. Most areas bordering structures and roadside ditches were well maintained, while unmanaged grasses/herbaceous vegetation and shrubs border ponds, streams, and some drainage ditches, providing limited space, cover, and food for small mammals.

The expansion site under development is bounded by Cp Humphreys and the Anseongcheon River. During 2008, areas immediately adjacent to Cp Humphreys were under development for the construction of structures for military operations and housing, roads, drainage systems, and recreation areas. A large area not under development consisted of low lying hills of grasses/herbaceous vegetation, small groves of trees, major irrigation/drainage systems to reduce flooding, rice paddies, and limited dry-land farmland that lay fallow with unmanaged grasses/herbaceous vegetation. The major irrigation/drainage systems were bordered by moderate/tall grasses along the banks extending to roadways that harbored relatively high small mammal populations. Grading and filling of the unmanaged fallow lands continued and by 2010 much of the expansion site had been graded and cleared of grasses/herbaceous vegetation in preparation for land fill and construction. Habitats of small mammals (e.g., rodents and soricomorphs) and predators (e.g., weasels, raccoon dogs, and feral cats) were destroyed as lands were sequentially graded and filled, leaving these areas devoid of vegetation except for limited areas of grasses/herbaceous vegetation bordering ditches, major drainage systems for flood control, and temporary roads that provided habitat for small mammals.

### Small mammal trapping

Trapping was conducted monthly from January 2008-December 2009, quarterly during 2010, and semiannually in 2012. Areas surveyed included: limited tall grasses/herbaceous vegetation that bordered streams, retaining ponds, fence lines, park perimeters, and open fields at Cp Humphreys and expansive tall grasses/herbaceous vegetation bordering dirt and paved roads, streams, ditches, water impoundments, flood control drainage systems, spillways, and the Anseongcheon River at the expansion site, which did not interfere with military or construction activities. Collapsible live-capture Sherman® traps (7.7x9x23 cm; H.B. Sherman, Tallahassee, FL) were set in grasses/herbaceous vegetation (providing shade) at 4–5 m intervals (25–50 traps/trap line) in the late afternoon over a 2–3 day period and picked up early the following morning as previously described [4, 6–10]. Traps positive for small mammals were sequentially numbered, placed in a secure container, and transported to Korea University where they were euthanized, identified to species using morphological techniques, sexed, weighed and then, tissues (lung, liver, kidney, and spleen) collected and stored at -80°C until used [14].

### Serological and molecular screening for hantaviruses

Small mammal sera were diluted 1:16 in phosphate buffered saline and examined for IgG antibodies against HTNV, SEOV, Prospect Hill virus, and Imjin virus (MJNV) by indirect immunofluorescent antibody test (IFAT) [15–18]. Lung tissues of hantavirus Ab+ rodents and soricomorphs were used for the identification of the hantavirus gene by RT-PCR that amplified a portion of the G_C_ glycoprotein-encoding M segment. Total RNA, extracted from lung tissues of the seropositive animals using the RNA-Bee Kit (TEL-TEST Inc., Friendswood, TX), was reverse-transcribed using Superscript® II RNase H-reverse transcriptase kit (Invitrogen, Carlsbad, CA) according to the manufacturer’s instructions. Nested-PCR using the primers (outer primer set, 5’-TGGGCTGCAAGTGC-3’, 5’-ACATGCTGTACAGCCTGTGCC-3’; inner primer set, 5’-TGGGCTGCAAGTGCATCAGAG-3’, 5’-ATGGATTACAACCCCAGCTCG-3’) was performed to recover a 373-nucleotide (nt) region of the hantavirus M segment [19–21]. Amplified products were size-fractionated by electrophoresis on 1.5% agarose gels containing ethidium bromide (0.5 mg/mL). The PCR products were cloned using the pST Blue-1 vector (Novagen, Dormstadt, Germany) and plasmid DNA purified using the QIAprepSpinMiniprep kit (QIAGEN Inc., Chatsworth, CA). DNA sequencing was performed in both directions from at least three clones of each PCR product using the Big-Dye® Terminator v3.1 cycle sequencing kit (Applied Biosystems, Foster City, CA) on an automated sequencer (Model 3730, Applied Biosystems).

### Real-time quantitative PCR (RT-qPCR)

cDNA was synthesized using a High Capacity RNA-to-cDNA kit (Applied Biosystems) by adding 1 μg of total RNA from lung tissues of HTNV-positive *A*. *agrarius*. RT-qPCR was conducted using SYBR Green PCR Master mix (Applied Biosystems) on a StepOne Real-Time PCR System (Applied Biosystems). The primer sequences included a forward primer; 5’-TTATTGTGCTCTTCATGGTTGC-3’ and a reverse primer; 5’-CATCCCCTAAGTGGAAGTTGTC-3’ for HTNV S segment [22]. The cycling program was a cycle of 95°C for 10 min, followed by 40 cycles of 15 s at 95°C and 1 min at 60°C.

### Whole genome sequencing of HTNV

Total RNA was isolated from lung tissues of HTNV-positive *A*. *agrarius* using a Hybrid R Kit (GeneAll Biotechnology, Seoul, ROK). cDNA was synthesized using High Capacity RNA-to-cDNA kit (Applied Biosystems) with random hexamer or 5′-TAGTAGTAGACTCC-3′. Using Ex Taq DNA polymerase (TaKaRa BIO Inc., Shiga, Japan), first and second RT-PCR were performed at 95°C for 4 min, followed by 6 cycles of denaturation at 94°C for 30 sec, annealing at 37°C for 30 sec, elongation at 72°C for 1 min 30 sec, and then 32 cycles of denaturation at 94°C for 30 sec, annealing at 42°C for 30 sec, and elongation at 72°C for 1 min 30 sec, and 72°C for 5 min (ProFlex PCR System, Life Technology, CA, USA). To complete whole genome sequencing of HTNV, rapid amplification of cDNA ends (RACE) for 3’ and 5’ termini were performed using 3’ and 5’-Full RACE Core Sets (Takara, Shiga, Japan) according to manufacturer specifications.

### Genetic and phylogenetic analyses

Alignments of whole genome sequences of HTNV L, M, and S segments were facilitated using the Clustal W method (Lasergene program version 5, DNASTAR Inc. Madison, WI). The phylogenetic tree was generated by neighbor joining (NJ) and maximum likelihood (ML) methods (Molecular Evolutionary Genetics Analysis, 6.0). Genetic distances were computed, and topologies evaluated by bootstrap analysis of 1,000 iterations [23].

## Results

### Capture rates for species

A total of 2,364 (capture rate = 20.92%) small mammals over 11,300 trap nights, belonging to the Orders Rodentia [Families Muridae (3 species) and Cricetidae (3 species)] and Soricomorpha [Family Soricidae (1 species)], were captured monthly from 2008–2009, quarterly during 2010, and biannually during 2012 at Cp Humphreys (11.91%) and the expansion site (28.88%) **(Table 1)**.

**Table 1:** Number, capture rate, and percent of small mammals captured, by species, at both Camp Humphreys and the expansion site (under development), monthly 2008–2009, quarterly 2010, and biannually 2012.

Location^a^ (# Traps)^b^	Species	*Apodemus agrarius*	*Microtus fortis*	*Micromys minutus*	*Mus musculus*	*Myodes regulus*	*Rattus norvegicus*	*Crocidura lasiura*	*Tscherskia triton*	Total
Camp Humphreys (5,300)	Number Captured	510	34	12	25	1	0	49	0	631
	Capture Rate	9.62	0.64	0.23	0.47	0.02	0.00	0.92	0.00	11.91
	Percent Captured	80.82	5.39	1.90	3.96	0.16	0.00	7.77	0.00	
Expansion Site (6,000)	Number Captured	1,302	220	29	42	1	3	135	1	1,733
	Capture Rate	21.70	3.67	0.48	0.70	0.02	0.05	2.25	0.02	28.88
	Percent Captured	75.13	12.69	1.67	2.42	0.06	0.17	7.79	0.06	
Total (11,300)	Number Captured	1,812	254	41	67	2	3	184	1	2,364
	Capture Rate	16.04	2.25	0.36	0.59	0.02	0.03	1.63	0.01	20.92
	Percent Captured	76.65	10.74	1.73	2.83	0.08	0.13	7.78	0.04	

^a^Preexisting Camp Humphreys installation and the projected expansion site under construction (grading and land fill of rice paddies).

^b^Number of traps set at Camp Humphreys and the expansion site.

Overall, *A*. *agrarius* (striped field mouse) accounted for 76.65% (1,812) of all small mammals captured, followed by *Microtus fortis* (reed vole) (254, 10.74%), *Crocidura lasiura* (Ussuri white-toothed shrew) (184, 7.78%), *Mus musculus* (house mouse) (67, 2.83%), *Micromys minutus* (harvest mouse) (41, 1.73%), *Rattus norvegicus* (brown rat) (3, 0.13%), *Myodes regulus* (Royal or Korean red-backed vole) (2, 0.08%), and *Tscherskia triton* (greater long-tailed hamster) (1, 0.04%). The overall capture rate of *A*. *agrarius* for unmanaged tall grass habitats at the expansion site were higher (21.70%) than for limited tall grass habitats at Cp Humphreys (9.62%) **(S1 Fig)**. *A*. *agrarius* accounted for 80.82% and 75.13% of all small mammals at Cp Humphreys and the expansion site, respectively. The lower proportion of *A*. *agrarius* captured at the expansion site was due to large numbers of canals and flooded fallow rice paddies that are primary habitats for *M*. *fortis*.

### Capture rates for years and months

Annual capture rates for *A*. *agrarius* for both Cp Humphreys and the expansion site for years 2008–2009 were more than 4-fold and 3-fold higher, respectively, than for years 2010 and 2012, when a large portion of unmanaged vegetation was cleared at the expansion site **(Table 2, S2 Fig)**.

**Table 2:** Annual capture rates for small mammals, by species, at Camp Humphreys and the expansion site, captured monthly during 2008–2009, quarterly 2010, and biannually 2012.

Collection Site	Year (No. Traps)	*Apodemus agrarius*	*Microtus fortis*	*Micromys minutus*	*Mus musculus*	*Myodes regulus*	*Rattus norvegicus*	*Crocidura lasiura*	*Tscherskia triton*	Total
Camp Humphrey^a^	2008 (1,600)	14.31	0.75	0.44	0.00	0.00	0.00	1.81	0.00	17.31
	2009 (2,400)	9.17	0.75	0.21	0.58	0.00	0.00	0.58	0.00	11.29
	2010 (1,000)	4.90	0.40	0.00	0.00	0.00	0.00	0.60	0.00	5.90
	2012 (300)	2.00	0.00	0.00	1.83	0.17	0.00	0.00	0.00	4.00
Expansion Site^b^	2008 (2,050)	29.66	0.93	0.73	0.24	0.05	0.00	1.41	0.00	33.02
	2009 (2,475)	25.01	7.84	0.36	1.29	0.00	0.12	4.28	0.00	38.91
	2010 (1,175)	4.68	5.61	0.50	0.26	0.00	0.00	0.00	0.00	5.87
	2012 (300)	6.67	0.33	0.00	0.67	0.00	0.00	0.00	0.33	8.00
Overall Mean	2008 (3,650)	22.93	0.85	0.60	0.14	0.03	0.00	1.59	0.00	26.14
	2009 (4,875)	17.21	4.35	0.29	0.94	0.00	0.06	2.46	0.00	25.31
	2010 (2,175)	4.78	0.46	0.23	0.14	0.00	0.00	0.28	0.00	5.89
	2012 (600)	5.33	0.17	0.00	2.17	0.17	0.00	0.00	0.17	8.00

^a^Preexisting Camp Humphreys installation where uncut tall grasses/ herbaceous vegetation and shrubs predominated along ditches, streams, and water impoundment areas.

^b^Expansion site under development (clearing vegetation, grading and land fill) and construction of structures for housing and military operations, roads, drainage systems, and recreational areas.

**Fig 2:**
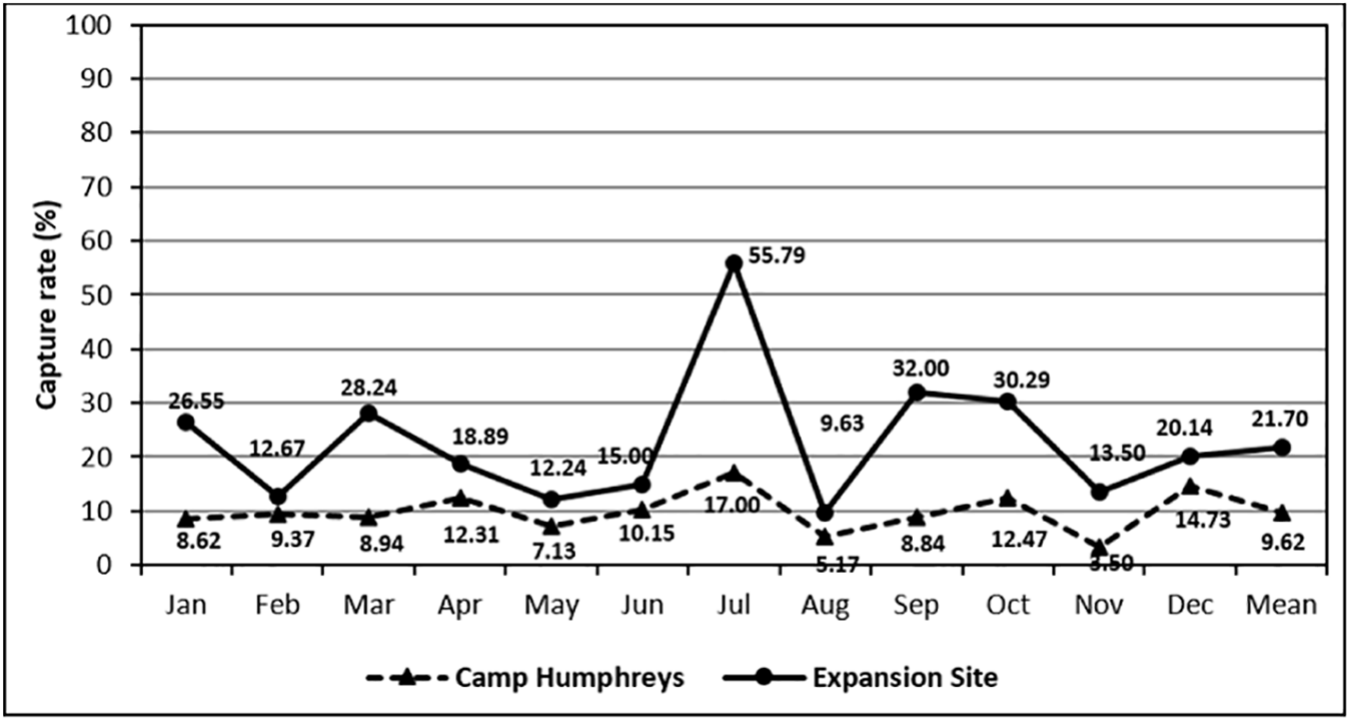
Monthly capture rates of *Apodemus agrarius* collected at Camp Humphreys and the new expansion site from 2008–2010 and 2012.

Monthly capture rates for *A*. *agrarius* varied for both Cp Humphreys (range 3.50–17.00%) and the expansion site (range 9.63–55.79%) **(Fig 2)**.

### Capture rates for sexes

*Apodemus agrarius* capture rates for males (46.69%, range 33.33–55.56%) and females (53.31%, range 44.44–66.67%), were not significantly different (*p* = 0.062) **(Table 3)**. Gravid females were only observed from April-November, with the highest gravid rates observed during June (40.00%), August (41.67%), and September (45.16%) and ranged from 9.43–16.21 for the other months (Table 3).

**Table 3:** The total number and percentage of *Apodemus agrarius* males and females captured and number females (%) gravid at Camp Humphreys and the new expansion site, monthly from 2008–2009, quarterly 2010, and biannually 2012.

Month	No. Males	(%)	No. Females	(%)	No. Gravid	(%)
January	41	40.59	60	59.40	0	0
February	98	50.00	98	50.00	0	0
March	74	46.83	84	53.16	0	0
April	51	40.80	74	59.20	12	16.21
May	61	42.96	81	57.04	13	16.05
June	48	51.61	45	48.39	18	40.00
July	164	54.85	135	45.15	21	15.56
August	60	55.56	48	44.44	20	41.67
September	77	45.29	93	54.71	42	45.16
October	53	33.33	106	66.67	10	9.43
November	17	50.00	17	50.00	2	11.76
December	102	44.93	125	55.07	0	0
TOTAL	846	46.69	966	53.31	138	14.29

### Capture rates for weights

Few *A*. *agrarius* weighed ≤10 g (0.61%) or >40 g (2.37%), with most weighing 10–20 g (39.96%), 20–30 g (37.97%) and 30–40 g (19.09%) **(Table 4)**.

**Table 4:** The total number and percentage of male and female *A*. *agrarius* captured, by weight category, mean weights for each category, and differences of mean male weights—Mean female weights for each weight category during 2008–2010 and 2012.

Categories	≤10 g	10–20 g	20–30 g	30–40 g	>40 g	Total
Number (%)^a^ ♂	6 (54.54)	244 (33.70)	315 (45.78)	242 (69.94)	38 (88.37)	845 (46.63)
Number (%)^a^ ♀	5 (45.45)	480 (66.30)	373 (54.21)	104 (30.06)	5 (11.63)	967 (53.37)
Total (♂,♀)^b^	11 (0.61)	724 (39.96)	688 (37.97)	346 (19.09)	43 (2.37)	1,812
Mean Weight^c^ ♂	9.3	17.0	24.7	34.2	43.7	25.5
Mean Weight ♀	8.8	16.3	25.3	33.5	42.9	23.2
Difference (♂-♀)	+0.5	+0.7	-0.6	+0.7	+0.8	+2.3

^a^Percent males or females = number males or females captured for each weight category/total number of males or females collected for each weight category, respectively.

^b^Percent = total number of males and females captured by weight category/total number captured.

^c^Mean weights = total weight of all specimens, by weight category/total number of specimens for each category.

Males accounted for 69.94% and 88.37% of those weighing 30–40 g and >40 g (old rodents), while females accounted for 66.30% and 54.21% of those weighing 10–20 g and 20–30 g (young rodents), respectively. On the average, male *A*. *agrarius* weighed 0.4–0.8 g more than females for each weight category, except for those weighing 20–30 g where females was heavier by 0.6 g more than the males.

Seasonal variations in population weights coincided with reproductive activity, e.g., increased proportions of lower weight individuals following high gravid rates **(Table 5)**.

**Table 5:** Quarterly total number and percentage (%) of *A*. *agrarius* captured, by weight category, at Camp Humphreys and the expansion site from 2008–2010 and 2012.

Trapping Season	Weight Class (g)					Total (%)
	≤ 10	10–20	20–30	30–40	>40	
Winter (Jan-Mar)	0	274 (60.21)	159 (34.95)	21 (4.61)	1 (0.22)	455 (25.11)
Spring (Apr-Jun)	4 (1.11)	92 (25.56)	202 (56.11)	57 (15.83)	5 (1.38)	360 (19.86)
Summer (Jul-Sep)	5 (0.87)	86 (14.90)	224 (38.82)	230 (39.86)	32 (5.55)	577 (31.84)
Fall (Oct-Dec)	2 (0.48)	272 (64.76)	103 (24.52)	38 (9.05)	5 (1.19)	420 (23.18)
TOTAL (%)	11 (0.61)	724 (39.96)	688 (37.97)	346 (19.09)	43 (2.37)	1,812

**Fig 3:**
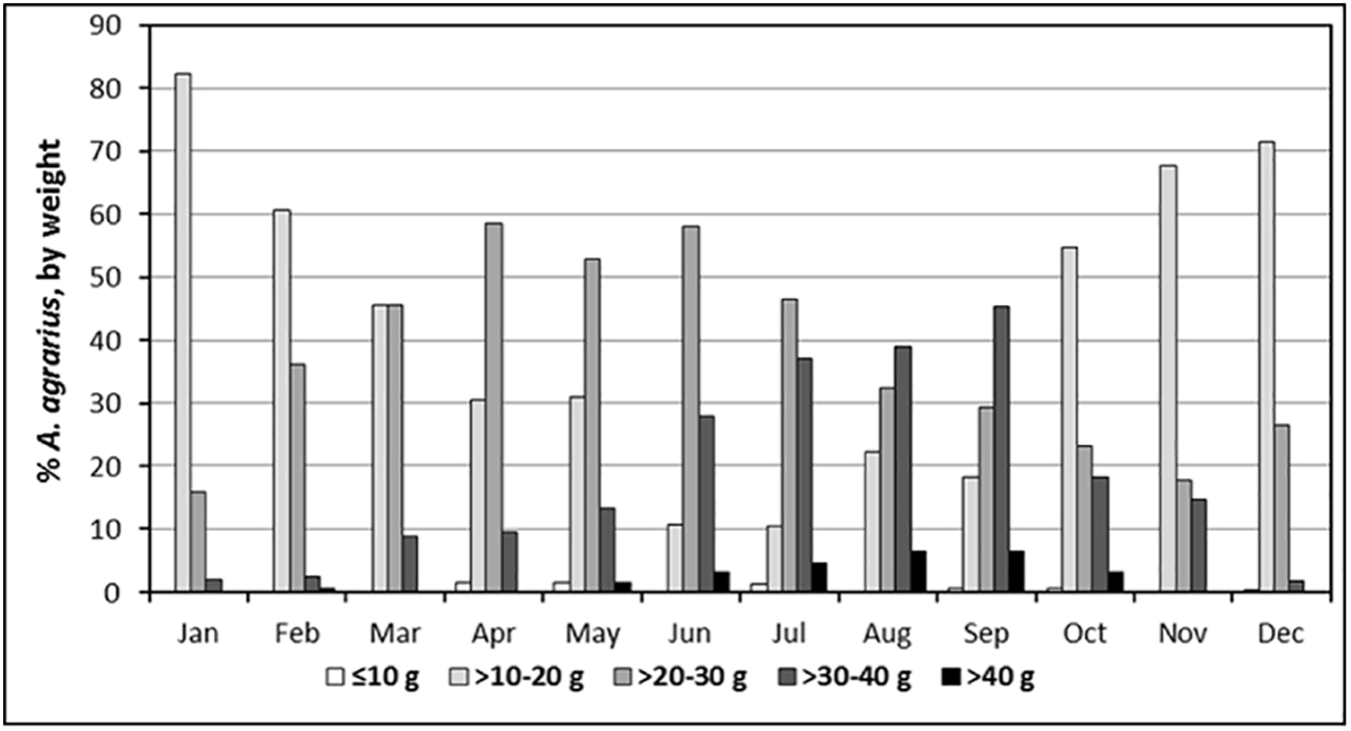
Overall percentage of *Apodemus agrarius* captured monthly, by weight, at Camp Humphreys and the new expansion site from 2008–2010 and 2012.

The proportion of *A*. *agrarius* weighing ≤20 g declined from a high of 82.18% in January to a low of 10.37% by July before increasing to a high of 71.37% by December **(Fig 3)**. In contrast, the proportion of *A*. *agrarius* weighing >30 g increased from a low of 1.98% in January to a high of 51.76% by September as a result of a maturing populations, gravid females, and abundant food supply, but rapidly declined to 1.76% by December that followed high numbers of gravid females during August (41.67%) and September (45.16%).

### Serological prevalence of HTNV at Cp Humphreys and the expansion site, Pyeongtaek

**Fig 4:**
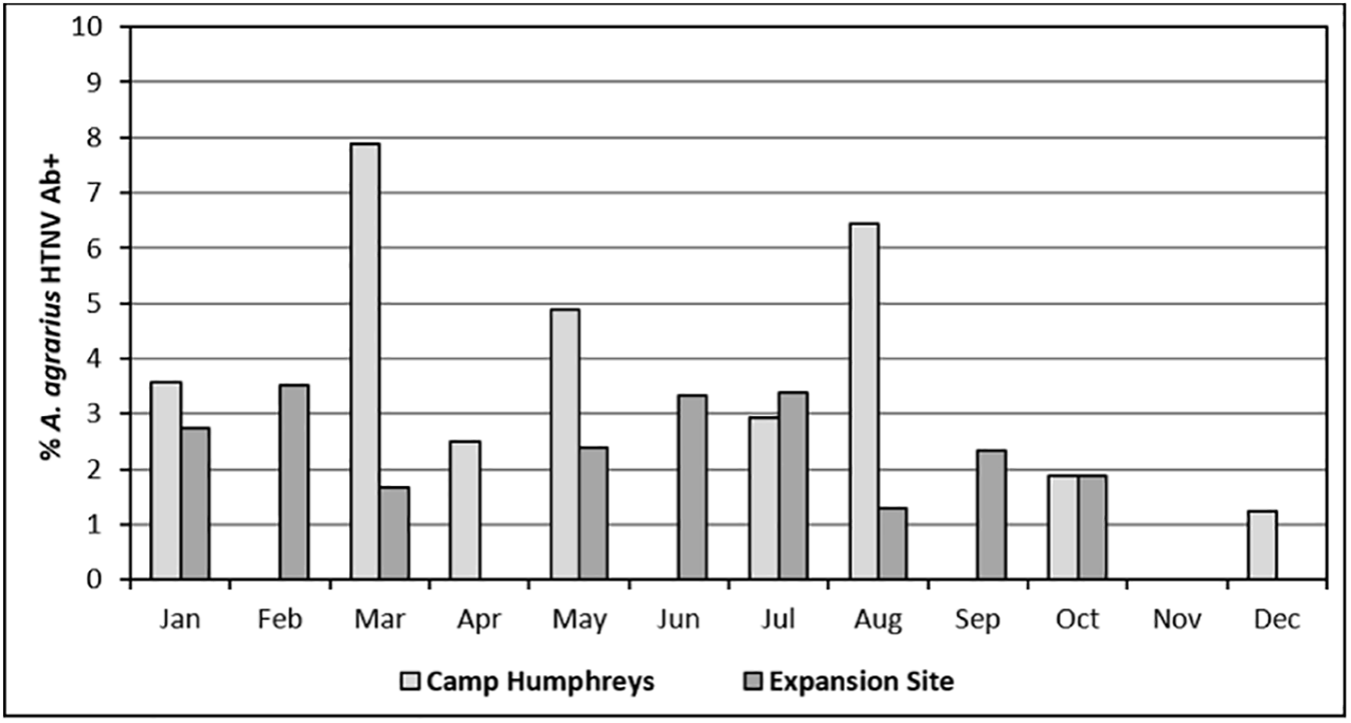
Overall percentage of *Apodemus agrarius* captured monthly at Camp Humphreys and the new expansion site that were antibody-positive for Hantaan virus.

Among 1,812 *A*. *agrarius* captured at Cp Humphreys and the expansion site, IFAT showed that 39 (2.15%) rodents were positive for anti-HTNV IgG. HTNV Ab+ rates among *A*. *agrarius* were significantly higher for Cp Humphreys (2.41%, range 0.0–7.89%) than for the expansion site (2.11%, range 0.0–3.51%) (χ^2^ = 17.279, df = 1, P<0.001) **(Fig 4)**. However, based on trap rates (Cp Humphreys, 9.62; expansion site, 21.70), the number of HTNV Ab+ *A*. *agrarius* captured/100 traps was 2-fold greater at the expansion site (0.46) compared to Cp Humphreys (0.23). The quarterly seasonal proportions of HTNV Ab+ *A*. *agrarius* for all weight categories at Cp Humphreys and the expansion site varied from 0.95% (Oct-Dec) to 2.86% (Jan-Mar) **(Table 6)**.

**Table 6:** Quarterly number (%) of *A*. *agrarius* (total number HTNV Ab+/total number captured, by weight category, at Camp Humphreys and the expansion site, Pyeongtaek, Gyeonggi Province, 2008–2010 and 2012.

Trapping Season	Weight Class (g)					Total (%)
	≤ 10	10–20	20–30	30–40	>40	
Winter (Jan-Mar)	0	8/274 (2.92)	5/159 (3.14)	0/21 (0.0)	0/1 (0.0)	13/455 (2.86)
Spring (Apr-Jun)	0/4 (0.0)	0/92 (0.0)	5/202 (2.48)	1/57 (1.75)	0/5 (0.0)	6/360 (1.67)
Summer (Jul-Sep)	0/5 (0.0)	2/86 (2.33)	4/224 (1.79)	6/230 (2.61)	4/32 (12.50)	16/577 (2.77)
Fall (Oct-Dec)	0/2 (0.0)	1/272 (0.37)	2/103 (1.94)	1/38 (2.63)	0/5 (0.0)	4/420 (0.95)
TOTAL (%)	0/11 (0.0)	11/724 (1.52)	16/688 (2.33)	8/346 (2.31)	4/43 (9.30)	39/1,812 (2.15)

**Fig 5:**
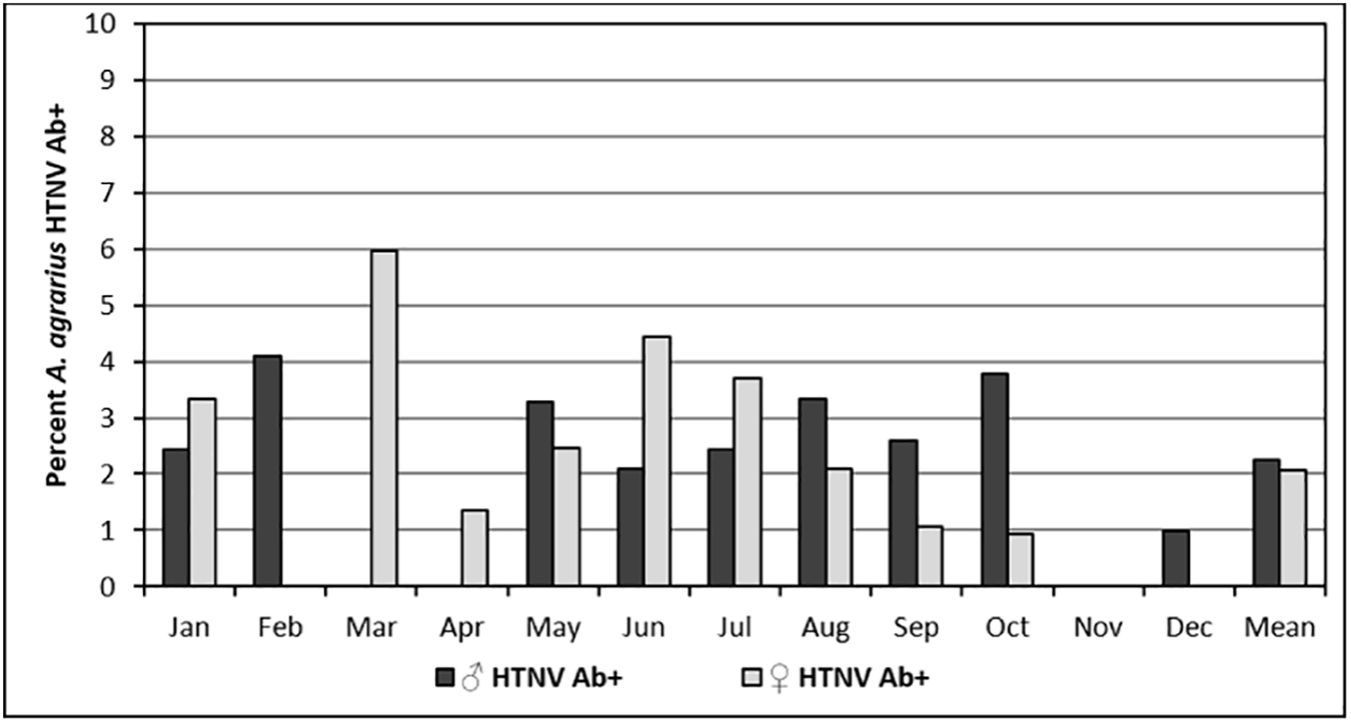
Overall monthly percentage of male and female *Apodemus agrarius* captured at Camp Humphreys and the new expansion site that were antibody-positive for Hantaan virus, 2008–2010 and 2012.

**Fig 6:**
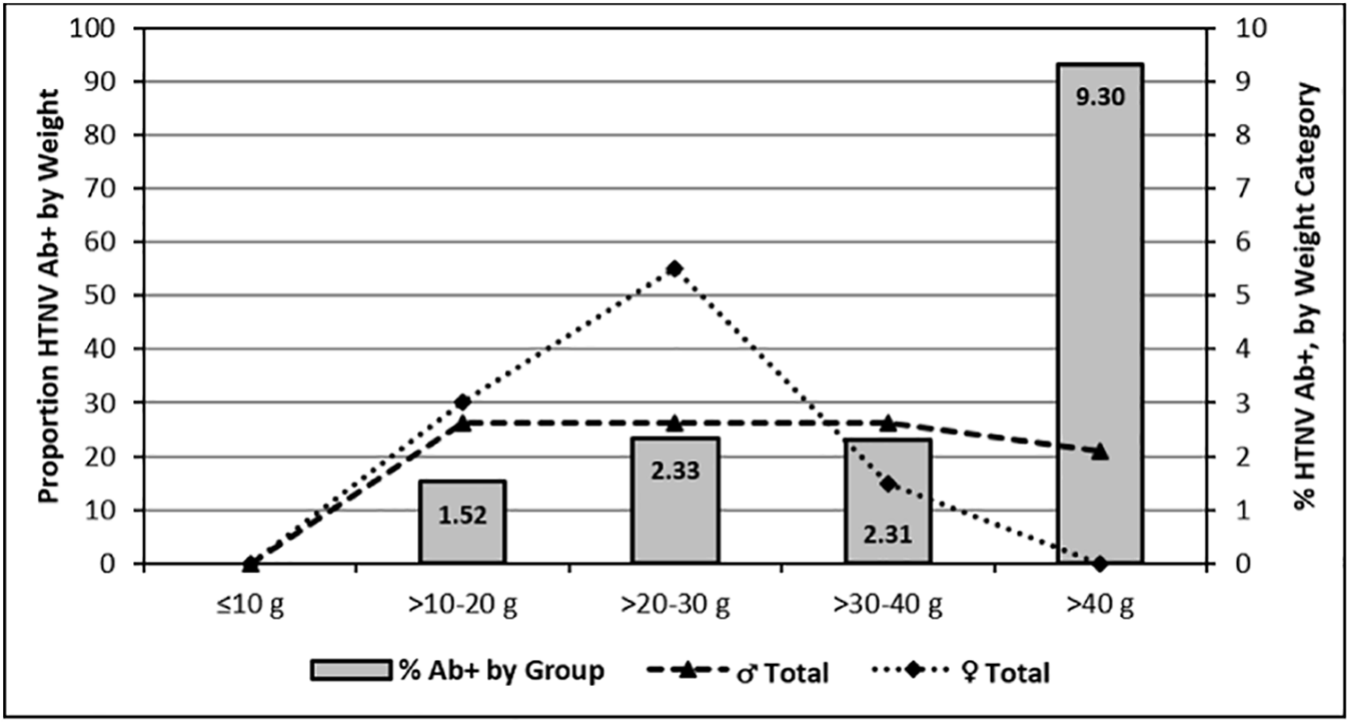
Overall percent of *Apodemus agrarius*, by sex, captured at Camp Humphreys and the new expansion site that were HTNV Ab+ for each weight category (lines) and percent HTNV Ab+ within each weight category (bars).

Overall, monthly HTNV Ab+ rates for males (2.25%) and females (2.07%) were similar, but varied monthly from 0.0–4.08% for males and 0.0–5.95% for females **(Fig 5)**. The proportion of *A*. *agrarius* males and females that were serologically positive for HTNV for weight categories 10–20 g (1.52%), 20–30 g (2.33%), and 30–40 g (2.31%) were similar **(Fig 6)**. However, the serological positivity (9.30%) of HTNV Ab for *A*. *agrarius* weighting > 40 g (oldest rodents) was only observed in males and was significantly higher than the other weighted groups (One-way ANOVA test, p<0.0001).

### Molecular prevalence of HTNV at Cp Humphreys and the expansion site, Pyeongtaek

To identify HTNV RNA in the seropositive *A*. *agrarius*, RT-PCR was performed by targeting the G_C_ glycoprotein-encoding M segment (373 bps). A total of 11 (28.21%) *A*. *agrarius* harbored HTNV RNA in 39 HTNV Ab+ rodents. The molecular prevalence of HTNV for males and females was 26.32% (5/19) and 30.00% (6/20), respectively **(Table 7)**.

**Table 7:** Serological and molecular prevalence of HTNV from *A*. *agrarius* captured, by sex, weight, season category, at Camp Humphreys and the expansion site from 2008–2010 and 2012.

Categories	Seropositive rate for anti-HTNV IgG antibody (%)	HTNV RNA positivity (%)
Sex (n = 1,812)		
Males	19/845 (2.25)	5/19 (26.32)
Females	20/967 (2.07)	6/20 (30.00)
Weight (n = 1,812)		
≤10 g	0/11	0
10–20 g	11/724 (1.52)	2/11 (18.18)
20–30 g	16/688 (2.33)	6/16 (37.50)
30–40 g	9/346 (2.60)	2/9 (22.22)
>40 g	3/43 (6.98)	1/3 (33.33)
Season (n = 1,812)		
Winter (Jan-Mar)	7/455 (1.54)	4/7 (57.14)
Spring (Apr-Jun)	20/360 (5.56)	3/20 (15.00)
Summer (Jul-Sep)	8/577 (1.39)	2/8 (25.00)
Fall (Oct-Dec)	4/420 (0.95)	2/4 (50.00)

The proportion of HTNV-positive *A*. *agrarius* for weight categories 10–20 g (18.18%), 20–30 g (37.50%), 30–40 g (22.22%), and 40 g (33.33%) was similar. Seasonal positivity of HTNV in *A*. *agrarius* showed 57.1% (Jan-Mar), 15% (Apr-Jun), 25% (Jul-Sep), and 50% (Oct-Dec).

**Fig 7:**
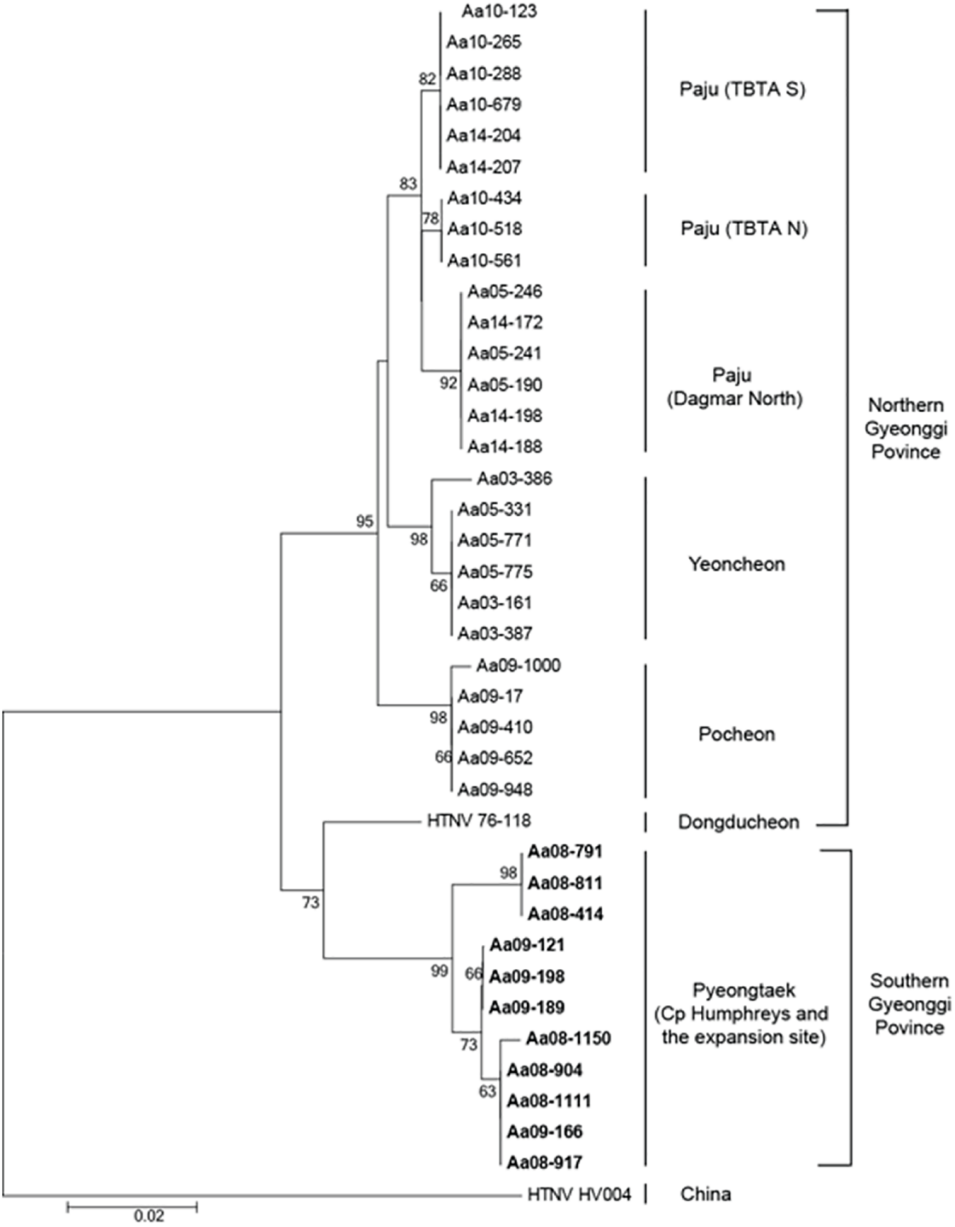
Phylogenetic analysis of Hantaan virus strains identified in Cp Humphreys and the new expansion site, Pyeongtaek, based on a 328-nt region of the G_C_ glycoprotein-encoding M segment. The phylogenetic tree was generated by Neighbor-joining (NJ) method. Branch lengths are proportional to the number of nucleotide substitutions, while vertical distances are for clarity. The numbers at each node are bootstrap probabilities (expressed as percentages), as determined for 1000 iterations (GenBank accession numbers; KX119152-119162).

The partial sequences of HTNV M segment (328nt length) were trimmed and used for analysis. All HTNV strain sequences were submitted to GenBank (Accession numbers; KX119152-119162). The partial M segment sequences (coordinates 1,994 to 2,321) of 11 HTNV strains from Cp Humphreys and the expansion site were phylogenetically compared to HTNV strains previously identified in military training sites, northern Gyeonggi province **(Fig 7)**. The nucleotide and amino acid homologies of the 11 HTNV strains from Cp Humphreys and the expansion site varied between 0–3.1% and 0–2.8%, respectively.

### Quantitative RT-PCR, whole-genome sequencing, and phylogenetic analyses

**Fig 8:**
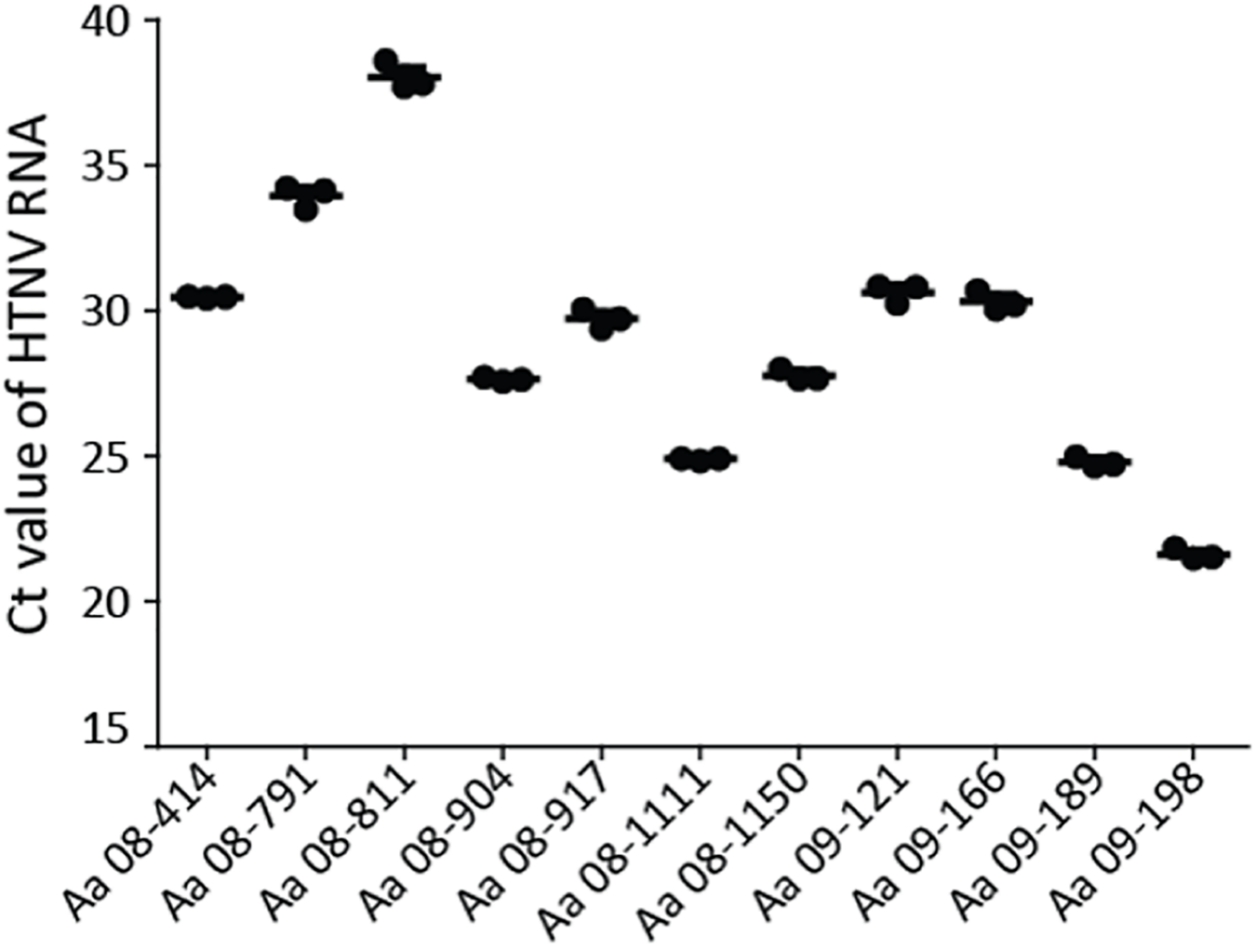
Determination of threshold cycle (Ct) values of Hantaan virus RNA in HTNV-positive *Apodemus agrarius* collected at Camp Humphreys and the new expansion site. HTNV RNA was examined for the HTNV S segment in anti-HTNV IgG seropositive and HTNV RNA positive rodents (n = 11). The vertical axis represents the Ct value of HTNV S segment RNA.

To obtain whole genome sequences of HTNV in serological and molecular positive *A*. *agrarius* from Cp Humphreys and the extension region, Pyeongtaek, HTNV RNA copies were quantified in the lung tissue by RT-qPCR. The threshold of Cycle (Ct) value are shown in the Fig 8. Aa09-198 demonstrated the lowest Ct value (highest viral loads), followed by Aa08-1111 and Aa09-189. The whole genome sequences of the three HTNV strains were recovered by conventional RT-PCR and RACE PCR for both 3’ and 5’ end sequences. The whole genome sequences of the HTNV strains deposited in GenBank (Accession numbers; KY594712- KY594720).

**Fig 9:**
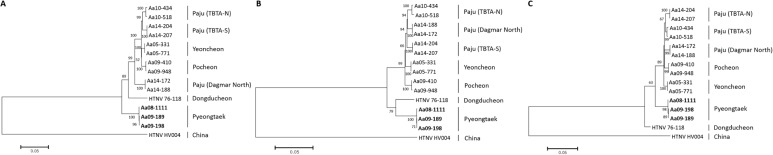
Phylogenetic analyses of the whole genome sequences of Hantaan virus L, M, and S segments identified at Camp Humphreys and the new expansion site, Pyeongtaek, Gyeonggi province. The phylogenetic tree was generated by Maximum-likelihood (ML) method. The phylogeny of the L segment (a), M segment (b), and S segment (c) is described. Branch lengths are proportional to the number of nucleotide substitutions, while vertical distances are for clarity. The numbers at each node are bootstrap probabilities (expressed as percentages), as determined for 1000 iterations. The phylogenetic positions of HTNV strains are shown in relationship to representative Aa10-434 (L segment, KT934970; M segment, KT935004; S segment, KT935038), Aa10-518 (L segment, KT934971; M segment, KT935005; S segment, KT935039), Aa14-204 (L segment, KT934977; M segment, KT935011; S segment, KT935045), Aa14-207 (L segment, KT934978; M segment, KT935012; S segment, KT935046), Aa05-331 (L segment, KT934962; M segment, KT934996; S segment, KT935030), Aa05-771 (L segment, KT934963; M segment, KT934997; S segment, KT935031), Aa09-410 (L segment, KU207177; M segment, KU207185; S segment, KU207193), Aa09-948 (L segment, KT934966; M segment, KT935000; S segment, KT935034), Aa14-172 (L segment, KT934974; M segment, KT935008; S segment, KT935042), Aa14-188 (L segment, KT934975; M segment, KT935009; S segment, KT935043), HTNV 76–118 (L segment, X55901; M segment, M14627; S segment, M14626) and HV004 (L segment, JQ083393; M segment, JQ083394; S segment, JQ083395).

The genetic diversity and phylogenetic relationship of HTNV in Cp Humphreys and the extension region were determined in comparison to strains obtained from lung tissue of seropositive rodents previously captured at a variety of HFRS-endemic areas, e.g. Twin Bridge Training Area (TBTA) North, TBTA South, and Dagmar North in Paju, Yeoncheon, and Pocheon **(Fig 9)**. The L segment of the HTNV formed an independent outgroup of all of HTNV in Gyeonggi province. The phylogenetic analysis of the M segment showed a well-supported genetic lineage with HTNV 76–118. The S segment formed a geographic-specific group within HTNV strains, including HTNV 76–118, in Gyeonggi province.

## Discussion

Cp Humphreys was designated a major US military hub with an estimated final US military and civilian population of >20,000 personnel. To accommodate for the increased population and expansion for military operations and outdoor recreational areas, adjacent lands that consisted mostly of low-lying rice paddies were purchased. The resulting environmental modifications of purchased properties included sequential grading of terrain and filling of low-lying fallow rice paddies for the construction of roads, ditches, major drainage system/recreational areas, military housing, schools, hospital and medical clinics, and other structures designed for military operations

*Apodemus agrarius* is associated with unmanaged lands characterized by abundant grasses/herbaceous vegetation in rural areas, including military training sites [24–27]. Similar to this survey and other annual and multi-year surveys, *A*. *agrarius* was the most commonly collected small mammal at US and ROK operated military training sites and installation field environments [4,6–10]. Compared to the expansion site of unmanaged grasses during 2008–2009, rodent populations were much higher than for Cp Humphreys where habitat was often limited to narrow strips of unmanaged vegetation along drainage systems/holding ponds. While capture rates of *A*. *agrarius* associated with the expansion site were high and movement of large trucks that created dusts on dirt, gravel, and hardened roads, the transmission risks of HTNV were reduced by very low HTNV Ab+ rates. Although no cases were reported among US military and civilian personnel, there may have been cases among local contractors and truck drivers that we were not aware of since these cases were not reported through the military medical system.

The Korea Centers for Control and Prevention (KCDC) [28] reports approximately 400–500 cases of HFRS annually, which are mainly caused by HTNV and SEOV. HTNV is the most common causative agent of HFRS in rural areas of the ROK and is characterized by severe medical manifestations and high mortality rate (9.46%) among US military personnel in Korea with good quality medical care from 1986–2014 [2,4–15]. In Korea, human infections of HTNV among military members are usually associated with high populations of *A*. *agrarius* in field environments or mice-infested vacant buildings in combination with “dust-creating” activities (e.g., back-blast from artillery, convoy operations, and track and wheeled vehicle maneuvers/operations in field environments), while SEOV infections are usually associated with urban environments activities (e.g., dry sweeping or vacuuming rodent infested buildings) where *R*. *norvegicus* predominates [3,5]. While HFRS caused by HTNV infections poses a serious health threat in Korea, it is classified by the US National Medical Intelligence Center (NCMI) as a rare disease, frequently occurring is small clusters. The most recent cluster among US military personnel deployed to the ROK was observed in 2005, when three US soldiers acquired HFRS at TBTA associated with exposure of contaminated dusts in wheeled vehicle cabs (cavalry unit) [3]. During the same year, another HFRS case was acquired at Firing Point 60, Yeoncheon, associated with the back-blast of artillery. More recently (Nov., 2014) a single case was reported for a US soldier conducting convoy and driver’s training at Dagmar North when HTNV seropositive rates in *A*. *agrarius* were 19.3% (considered a HFRS high-risk area), 27–30 days post-exposure that preceded infection [29]. The epidemiology of these cases was only accomplished through comparative analysis of the HTNV RNA from HFRS patients and associated rodents where the soldiers had trained, as the HTNV varies geographically [3]. Since 1986, only one case of SEOV has been reported from a US Airman that was vacuuming a rat-infested building and who had a relatively mild case of HFRS [5]. *R*. *norvegicus*, the primary reservoir for SEOV, is routinely captured by the Department of Public Works near housing and other facilities at Cp Humphreys. These resources would provide risk analyses for SEOV risks among US populations residing or working in buildings infested with rats.

Similar to rodent-borne disease surveillance conducted at training sites near the DMZ and other US military installations, *A*. *agrarius* was the most frequently collected small mammal [4,6–10]. Low to moderate *A*. *agrarius* capture rates were reported for limited tall grass habitats at Cp Humphreys and were similar to capture rates observed at other installations, e.g., Osan, Gunsan, and Gwangju Air Bases (unpublished data). During 2008–2009, high capture rates were observed for expansive tall grasses/herbaceous vegetation habitats at the expansion site and were similar to capture rates observed for expansive tall grass habitats at US and ROK operated training sites near the DMZ [4,6–10]. However, trap rates were significantly lower during 2010 and 2012 following grading and removal of much of the vegetation from the landscape that provided food and harborage for small mammals. Additionally, in part, the decline may have been due to over predation as the predator populations (e.g., raccoon dogs, feral cats, weasels, and predatory birds) were pushed into space-limited habitats surrounded by farming activities and urban environments. Over time, predator populations will likely stabilize based on available food sources and small mammal populations may rebound to near previous levels for undisturbed areas.

HFRS risks are associated with a combination of factors, including: environmental, reservoir host bionomics, and types of human exposure. Overall, HTNV Ab+ rates for Cp Humphreys/expansion site and Osan Air Base (50 km south of Seoul and 20 km north of Pyeongtaek), are very low, usually ≤6%, when compared to US and ROK operated training areas near the DMZ where seasonal HTNV Ab+ rates varied up to 60% during monthly surveys and overall annual rates varied from 15% to 25% [4,7,9,10]. Limited surveys at Gunsan and Gwangju Air Bases, near the southern tip of the Korean Peninsula, were indicative of low small mammal populations, as well as none of the *A*. *agrarius* were HTNV Ab+ (TA Klein, personal communication). The reason for high HTNV Ab+ rates near the DMZ that decrease over distance to the tip of the peninsula is not understood, but may be related to reproductive behaviors. For training areas near the DMZ, there were observed low reproductive periods during the winter (0–0.3%), followed by a late spring increase in reproduction (4.2–24.6%) (Apr-May), low reproduction during the summer (0–1.3%), and very high reproduction in the late summer/early fall (27.3–70.0%) (Aug-Sep). A large influx of HTNV naïve mice observed at training areas near the DMZ during the fall/early winter periods when temperatures become cooler and habitat is shrinking as vegetation dies likely results in increased territorial disputes, wounding, and higher rates of HTNV transmission [30]. At Cp Humphreys and the expansion site, peak numbers of gravid females were observed earlier (June, 40.0%) and similarly in August and September (42.5 and 25.2%, respectively), while moderate numbers of gravid females were observed during the early spring (April/May, 16.0–16.2%), July (15.6%), and early winter (October/November, 9.4–11.8%) [4,6–10]. In the southern area, young naïve *A*. *agrarius* broods throughout the summer may reduce territorial disputes in the fall due to relatively sufficient habitat and food. This proposed decreased movement and competition of naïve young rodents at Cp Humphreys and the expansion site may impact negatively on rodent-to-rodent HTNV transmission and result in lower HTNV Ab+ rates than those observed at the military training areas located near the DMZ, northern Gyeonggi province [31]. Additionally, the greatly reduced numbers of *A*. *agrarius* during 2010 likely reduced the potential for acute infections and corresponding viral shedding during the late fall/early winter when the majority of HFRS cases are reported. Although gravid females were observed throughout the early spring and summer, similar to training areas near the DMZ, the overall age (based on weight) of the population increased through September before rapidly declining as a result of the influx of young naïve rodents during the late fall reproductive cycle. By January, much of the population (based on weight) was replaced by young mice born during the late fall, indicating that the life span of *A*. *agrarius* live is approximately one year [24].

The overall HTNV seropositive rates of Cp Humphreys were higher than observed for the extensive tall grass habitats for undisturbed fallow rice paddies of the expansion area. However, the numbers of HTNV seropositive mice/100 traps were nearly 2-fold greater for the expansion site compared to Cp Humphreys, thereby increasing HFRS risks associated with less disturbed and unmanaged lands. The movement of potentially contaminated soil and vegetation and soil covered concrete roads created the potential for contaminated dusts and HTNV infections, especially for truck drivers and construction site monitors and workers. HTNV risks, while present at Cp Humphreys, are very low as a result of hard surface roads and recreation sites with short-cut grasses in the center, greatly reducing HTNV reservoir host habitats.

In this study, 39 (2.15%) of 1,812 *A*. *agrarius* were HTNV seropositive. The partial genome sequence of HTNV M segment was identified from 11 (28.21%) rodent lung tissues of HTNV Ab+ samples. RT-qPCR results showed varied viral loads in both sero- and molecular positive samples. The whole genome sequences of HTNV tripartite RNA were obtained from Aa08-1111, Aa09-189, and Aa09-198 that contained higher number of HTNV RNA copies. The termini of 3’ and 5’ sequences were determined by RACE PCR. Both end sequences of HTNV L, M, and S segments contained a mismatch at 9^th^ and the noncanonical U-G pair at 10^th^ nucleotides, suggesting the incomplete complementarity as previously described [32]. The total length of HTNV L segment for Cp Humphreys and the expansion site, Pyeongtaek, was three nucleotides shorter (6,530nt) than that of HTNV 76–118, demonstrating the deletion of 5’-AUC-3’ at the 5’ end of the L segment. U at the 12^th^ nucleotide on the M segment was defined compared to that on the HTNV 76–118 M segment.

The phylogenetic analyses of HTNV strains from Cp Humphreys and the extension site demonstrated a greater diversity of the rodent-borne hantavirus; the L segment showed distinct outgroup from entire HTNV strains, previously described in Gyeonggi province. The M segment formed a genetic cluster with HTNV 76–118, while the S segment was a geographic lineage within HTNV strains in Gyeonggi province. The natural reassortment and recombination of HTNV tripartite RNA genomes were observed near DMZ areas, northern Gyeonggi province [29,33]. Thus, the phylogenetic position and characterization of HTNV in Pyeongtaek will be clarified when additional genomic sequences of HTNV are acquired in southern areas of Korean peninsula.

A total of 10 (5.43%) *C*. *lasiura* were positive for MJNV, which was identified from shrews distributed in ROK and China, and the sera do not cross react with other rodent-borne hantaviruses [17]. Recently, there was a report that African shrew-borne hantaviruses were likely to infect humans [34]. Whether MJNV in *C*. *lasiura* poses a human health threat remains to be investigated. A total of 2/254 (0.79%) *M*. *fortis* and 1/41 (2.44%) *M*. *minutus* were serologically positive for hantaviruses, which was likely the result of interspecies transmission of HTNV since tissues were negative for hantaviruses by RT-PCR.

In summary, the characterization of US military installations undergoing expansion, in combination with small mammal surveillance, provides epidemiological information for the relative abundance of reservoir populations, hantavirus Ab+ rates, and other bionomic and environmental factors that are necessary to identify potential HTNV transmission risks. These transmission risks combined with human activities and exposure, which can be applied for disease risk analyses, are essential to the process of developing strategies for disease prevention. Comprehensive and long term rodent-borne disease surveillance should be the goal of US military preventive medicine to not only identify changes in HFRS disease risks due to modification of feral lands, but subsequently to better understand HFRS disease risks to soldiers, civilians, and family members residing and/or working on the installation. The whole genome sequences of HTNV at Cp Humphreys and the extension site show a greater diversity of rodent-borne hantaviruses in the ROK. Taken together, these data provide the robust impact to increase our knowledge of military activities, environmental conditions, and the genetic diversity of HTNV that can be applied to strategies to improve land management, disease risk mitigation, and the understanding of hantavirology.

## Supporting information

S1 Fig Overall capture rates (%) for rodents and soricomorphs captured at Camp Humphreys and the new expansion site from 2008–2010 and 2012. (TIF)

S2 Fig Annual capture rates and overall mean capture rates for years 2008–2010 and 2012 (bars) for rodents and soricomorphs captured at both Camp Humphreys and the new expansion site. Numbers are the overall mean capture rates, by species, for all years (2008–2010 and 2012).
(TIF)
