# Supplementary figures and images for: Hantavirus surveillance and genetic diversity targeting small mammals at Camp Humphreys, a US military installation and new expansion site, Republic of Korea
Source: PLoS One. 2017 Apr 27;12(4):e0176514. doi: 10.1371/journal.pone.0176514 (PMC5407799; doi:10.1371/journal.pone.0176514)

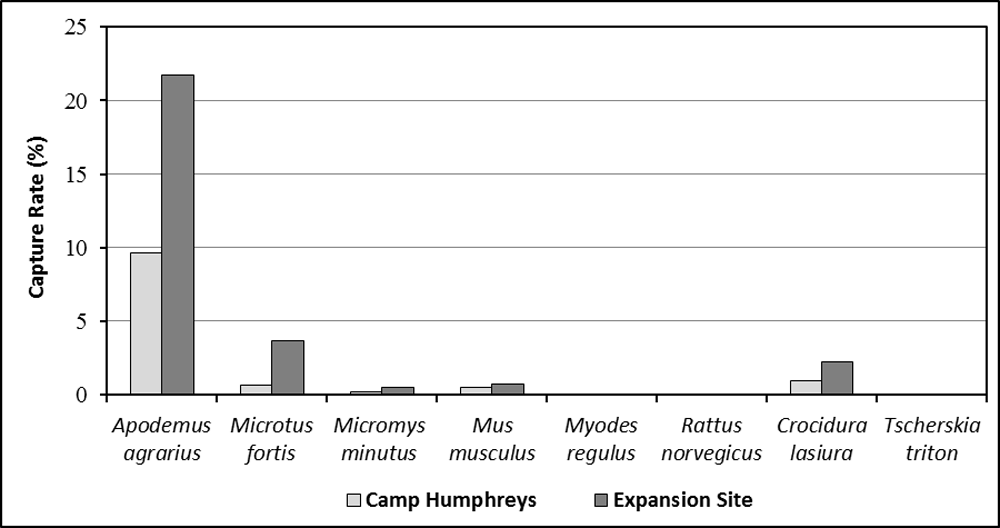

Supplement: S1 Fig — (TIF) [file pone.0176514.s001.tif]

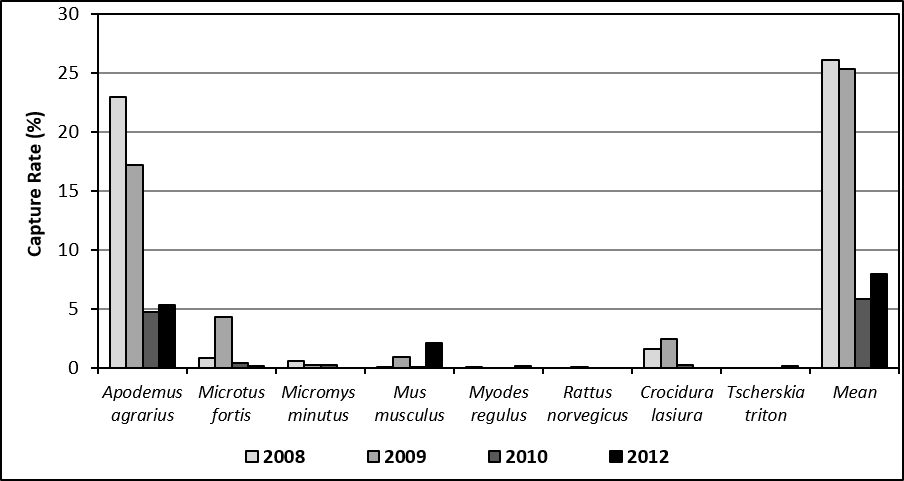

Supplement: S2 Fig — Numbers are the overall mean capture rates, by species, for all years (2008–2010 and 2012). (TIF) [file pone.0176514.s002.tif]
